# *Paederus* beetles: the agent of human dermatitis

**DOI:** 10.1186/s40409-015-0004-0

**Published:** 2015-02-25

**Authors:** Shabab Nasir, Waseem Akram, Rashad Rasool Khan, Muhammad Arshad, Iram Nasir

**Affiliations:** Department of Zoology, Wildlife and Fisheries, Government College University, Faisalabad, Pakistan; Department of Entomology, University of Agriculture, Faisalabad, Pakistan; Department of Statistics, Government College University, Faisalabad, Pakistan

**Keywords:** Dermatitis linearis, Irritation, Lesions, *Paederus*, Rove beetle, Skin dermatitis

## Abstract

**Background:**

Rove beetles of the genus *Paederus* cause dermatitis when they come in contact with human skin. This condition is prevalent in some tropical and subtropical regions, such as in northern Pakistan, where it was recorded for the first time by US troops. Despite much research from other countries on this subject, few studies, mostly clinical, have been performed in a Pakistani context. A survey was carried out in villages, towns and cities of Punjab province, Pakistan, to explore the rove beetle population dynamics and to develop a model to elucidate the symptoms, preventive measures and treatment strategies for this dermatitis.

**Methods:**

The prospective observational and patient surveys were performed bimonthly over a period of two years, in different districts of Punjab province. Collection was carried out in fields, gardens and houses during every visit with the aid of a pitfall trap, light trap, flight intercept trap, Berlese funnel trap and sweep netting. These traps were installed for four days during every visit. Interviews of ten individuals of different ages and sexes from each site were recorded during each visit.

**Results:**

Out of 980 individuals, 26.4% were found to suffer from *Paederus* dermatitis. Lesions were most commonly found on the neck followed by the face. In July-August during the rainy season, this skin irritation was most prevalent and the population of these beetles peaked (36.2%). During May-June, the beetle population was lowest (7.85%) due to soil dryness. About 70% of such irritation cases were from individuals living in farming villages or in farmhouses. Their houses typically (80%) had broken doors and screen-less windows while 97% of the residents were unaware of how they may have come into contact with these beetles. In most cases (91% from villages/small towns and 24% from cities and adjoining areas) the local residents were unaware of modern treatment strategies.

**Conclusions:**

*Paederus* dermatitis is extremely frequent in villages with poor housing facilities and could be avoided via community awareness.

## Background

*Paederus* dermatitis – also known as spider lick, night burn and dermatitis linearis – is a cutaneous condition that occurs due to physical contact with rove beetles belonging to the genus *Paederus* [1]. This is a specific form of irritant dermatitis often characterized by linear lesions on the exposed areas of the body, mainly on the neck and face, generally appearing during the night [1-5]. This irritation is not caused by a bite or sting, but rather by accidental brushing or crushing of a *Paederus* beetle over an exposed area of the human body [6]. Due to crushing, these beetles excrete a toxic substance in their hemolymph, which contains a vesicant called pederin that is more potent than cobra venom [7,8]. Pederin (C_25_ H_45_ O_9_ N) is an amide with two tetrahydropyran rings. This inhibits protein and DNA synthesis and hence stops cell division. Normally the palms of hands and the soles of feet are not affected by this chemical due to poor penetration [8].

*Paederus* dermatitis was first reported by Strickland in 1924. This skin irritation has been found most prevalent in the months from April to August [7,9]. The toxicity of *Paederus* has been reported in Western medicine for over 100 years and was also recognized in Chinese medicine 1200 years ago. More recently, *Paederus fuscipes* has been found to cause dermatitis in US troops in Afghanistan and northern areas of Pakistan [5,10].

Rove beetles belong to the Staphylinidae family, order Coleoptera. This family contains 3847 genera, including *Paederus*, which contains more than 622 known species [8,11]. Members of the genus *Paederus* are usually large and moderately convex. They are usually two-colored, with prominent black or blue and red coloration, but sometimes the entire body is a monochromatic black, blue or red. *Paederus fuscipes* Curtis, the most common species in our region, is the main cause of *Paederus* dermatitis. Its head is shiny black, its thorax and first four abdominal segments are red, its elytra are blue, and its last two abdominal segments are black. Its population increases markedly during the rainy season in July and August [12]. It is commonly found in agricultural crops, including maize, *bajra* (pearl millet) and berseem (Egyptian clover), as well as in domestic gardens [13]. This study was designed to elucidate the population dynamics of rove beetles and the peak season of *Paederus* dermatitis. A survey was also carried out to ascertain the demographic features and housing conditions of individuals living in the context of this cutaneous condition.

## Methods

This prospective observational patient survey study was performed over a period of two years, from January 2008 to December 2009 in several different districts of the Punjab province as shown in Table 1. The weather conditions are highly unpredictable in these districts (Summer 32 to 49°C and Winter 11 to −3°C). Both urban and rural areas were randomly selected. Rove beetles from selected areas were collected during every visit with the help of pitfall trap, light trap, flight intercept trap (FIT), Berlese funnel trap and sweep netting. As these beetles are found in a variety of habitats, different collection methods were used. Since these beetles are found running on land, a pitfall trap was used and because they are nocturnal and found in moist soils a Berlese funnel trap was also employed. A light trap was used due to their attraction towards light. These traps were installed for four days during every visit except sweep netting.

**Table 1:** **GPS positions of the selected localities**

**Plot no.**	**Locality name**	**Latitude**	**Longitude**	**Elevation (m)**
1	Lahore	31 14.287	73 59.513	194
2	Sheikhupur	31 34.723	73 29.117	187
3	Faisalabad	31 26.271	73 04.699	183
4	Multan	30 12.534	71 27.813	104
5	Rahim Yar Khan	28 26.450	70 19.712	83
6	Sargodha	32 05.379	72 40.566	183
7	Rawalpindi	33 34.425	73 05.161	496
8	D.G.Khan	30 18.209	70 43.324	117

The site visits were conducted at two-month intervals throughout the study period. The collected specimens were killed and preserved in 70% alcohol. The standard techniques for preserving and mounting were followed and included clearing in 10% KOH. Later the specimens were treated with glacial acetic acid and were dehydrated in ascending grades of alcohol and finally mounted in Hoyer’s medium. The specimens were identified with the aid of available taxonomic keys and experts (from Natural History Museum, Oslo) under a microscope in the laboratory of the Department of Zoology, Wildlife and Fisheries, Government College University, Faisalabad, Pakistan.

### Study design

A questionnaire was designed to ascertain the demographic characteristics (age, sex, farmer or non-farmer and education), individual knowledge of this cutaneous condition and living conditions of the people. The original language of the questionnaire was English but later this was translated into local languages according to the area, i.e., Punjabi, Urdu, Sraiki and Pothowari, and then the responses were retranslated into English. The Cronbach’s alpha value of the questionnaire was 0.745.

### Knowledge attitude and perception survey

The knowledge attitude and perception (KAP) survey was carried out from January 2008 to December 2009. One team of three individuals conducted interviews with one author, one local inhabitant of that area and one trained interviewer. As the primary option, questions were addressed to the head of the family as the main respondent. However, in the absence of a family head, another adult was interviewed. At least three attempts were made to interview the focal person of the family during the day (between 7:00 a.m. to 1:00 p.m.).

This cutaneous condition was evaluated by asking whether the respondent has any type of lesions on the body, and if so, the location and condition of those lesions; the respondent was then asked whether he or she takes any medicines, and if so, whether they were obtained through self-medication, a doctor or other source, and what type of lesions he or she had before and what might have been their origin. Furthermore, questions were asked about the respondent’s residence and bedroom (Where does the respondent live? Does he or she go to fields? Does he or she work in the fields? What are the number of rooms in the house, number of doors and windows in his or her room? Do the doors or windows provide proper closure? Are the doors and windows screened, and what is the source of light in the rooms?). The type of vegetation present around their houses was also noted. The age of the selected people ranged from 5 to 65 years but the patient age ranged from 14 to 45 years and included both sexes. All the subjects were arbitrarily selected from the streets of the villages and the cities. Every time the locality was same but the streets and the persons were either the same or different.

### Data management

Knowledge about the status of this cutaneous condition was defined in terms of the respondent’s information about the options in the questionnaire, namely the type of lesions, place of lesions, condition of lesions, if he or she gets medicine from a doctor or is self-medicated. Similarly, information about the living conditions of respondents, type of vegetation around their houses, information about the rooms, status of doors and windows in the rooms was also recorded bimonthly. The general scores for each respondent were between 0 and 3.

### Statistical analysis

The demographical characteristics were analyzed by the Chi-square test (to assess the association among factors and to calculate the p-value) and regression analysis (disease incidence versus beetle’s population) using Statistical Package of Social Science (SPSS 13). The remaining data were analyzed via simple percentage (%) and line graph.

## Results and discussion

### Demographic characteristics

The demographic characteristics of 980 people (444 urban and 536 rural) including males and females in a ratio of approximately 1:1 is shown in Table 2. The age of respondents varied from 3 to 65 years. The data showed non-significant differences in age and sex but highly significant discrepancies were recorded in education and work place (occupation).

**Table 2:** **Demographic characteristics of 980 respondents in urban and rural areas**

	**Urban no. (%)**	**Rural no. (%)**	**Total no. (%)**	**p value**
Gender				0.86
Male	247 (55.6)	301 (56.2)	548 (50.7)	
Female	197 (44.4)	235 (43.8)	432 (49.3)	
Age				0.09
<10	23 (5.1)	37 (6.9)	60 (6.1)	
10-20	107 (24.1)	95 (17.7)	202 (20.6)	
21-30	123 (27.7)	157 (29.3)	280 (28.6)	
31-50	154 (34.7)	189 (35.2)	343 (35)	
>50	37 (8.3)	58 (10.8)	95 (9.7)	
Education				≤0.001
Illiterate	32 (7.2)	76 (14.2)	108 (11)	
Upto middle	311 (70)	405 (75.5)	716 (73)	
Above middle	101 (22.8)	55 (10.3)	156 (16)	
Occupation				≤0.001
Farmers	124 (27.9)	435 (81.1)	559 (57)	
Non-farmers	320 (72.1)	101 (18.9)	421 (43)	

p-value indicates the association (strong or weak) among the factors.

Out of 980 observations, 259 (26.4%) were found to be affected with *Paederus* dermatitis. Out of the 259 affected individuals, 155 (60%) were female and 104 (40%) were male. A total of 70% of the victims were from farming communities living in the rural areas with no proper housing facilities (i.e. with broken doors, windows without screens) or they lived in the open air. The age of the patients ranged from 14 to 45 years with a mean age of 23.6 ± 4.7 years. A non-significant difference was found in the symptoms on the basis of body area or sex. Out of the 259 patients, 238 complained of burning sensations and 21 were asymptomatic. The majority (97%) of the affected subjects were not aware of this insect being the causative agent of their condition. The most commonly involved sites were the neck and face, as shown in Table 3. “Kissing” lesions were seen in ten cases (3.8%). However, significant differences were not found among different localities.

**Table 3:** **Parts of the body involved in lesions**

**Site**	**No. of patients with lesions**	**Percentage (%)**
Exposed	199	76.8
Neck	106	53.3
Face	59	29.6
Legs	24	12.1
Forearms	10	5.0
Covered	60	23.2
Shoulders	34	56.7
Thighs	26	43.3
Both (covered and exposed)	96	37

### Efficiency of trapping methods

**Figure 1:**
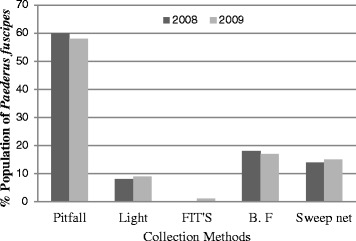
**Comparison of** ***Paederus fuscipes*** **fauna in different traps during 2008 and 2009.**

The greatest number of specimens (60and 58%) was collected with pit fall traps where as the least (0 and 1%) were collected by FITs during 2008 and 2009, respectively. Berlese funnel trap also contributed a significant portion (about 17%) in collection as shown in Figure 1. As these beetles generally move by running inspite of the ability to fly and are found in the soil or along the soil, they were mostly collected with a pitfall trap or Berlese’s funnel trap [14].

### Seasonal abundance of *Paederus fuscipes* and *Paederus* dermatitis

This skin irritation was reported mainly during March-April and July-August because the incidence of this dermatitis is directly related to the population of the beetle, especially *Paederus fuscipes*, which increases during these months. Subjecting the data to the analysis of variance revealed a significant relationship between disease incidence and beetle population as shown in Table 4. Furthermore, the regression equation (y = −4.55 + 1.403 x) showed that there was no chance of disease (y) in the absence of a beetle population (x), while every one unit increase in the beetle population (x) alters the disease incidence (y) by a factor of 1.403.

**Table 4:** **Regression analysis (ANOVA) revealing disease incidence versus beetle population**

**Source**	**Degree of freedom**	**Adjusted sum of squares**	**Adjusted mean of squares**	**f value**	**p value**
Regression	1	2924.24	2924.24	54.72	0.000
Error	10	534.43	53.44		
Lack-of-fit	8	526.43	65.80	16.45	0.059
Pure error	2	8.00	4.00		
Total	11	3458.67			

α = 0.05.

**Figure 2:**
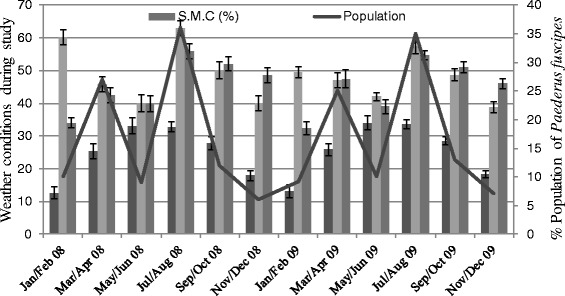
**The relationship between abiotic factors during different seasons from 2008 to 2009 and the population of** ***Paederus fuscipes.***

These insects require moist soils for their normal life cycle, so their population increased rapidly during March-April and July-August due to favorable abiotic environmental factors as shown in Figure 2 [9]. The difference in population during different months was due to biotic factors including different crops and such abiotic factors as temperature, relative humidity and soil moisture contents. These results are in accordance with the those of other scientists [15,16].

The symptoms of this disease occur principally on exposed parts of the body [1,9,16]. In our study we also found a predominance of lesions on the exposed areas (without any covering or clothes) of the body, mainly the face and neck. The results confirmed the presence of “kissing” lesions in the patients. These findings are consistent with similar studies conducted in Iran and Pakistan [9,17]. In our study, the majority of those afflicted were women because they worked in the fields cutting fodder from dawn to dusk. Most of this beetle population was found in agricultural areas producing different crops and fodders such as maize, wheat and Egyptian clover. These results corroborate the study of Bong *et al*. [15] that the crop type has significant effects on the activity of different staphylinid groups.

A potential contributing factor to this condition based on our study might be improper housing facilities. According to the respondents, mostly doors were broken and windows lacked screens, conditions under which the beetles might be frequently found indoors. Another factor contributing to this skin condition was the population’s ignorance of the causative agent, the behavior of this beetle, and potential precautionary measures. To address these shortcomings, we distributed the following information among the people:

During our study, we recorded the following symptoms that appeared or may appear on the patients’ skin:

### Symptoms

**Figure 3:**
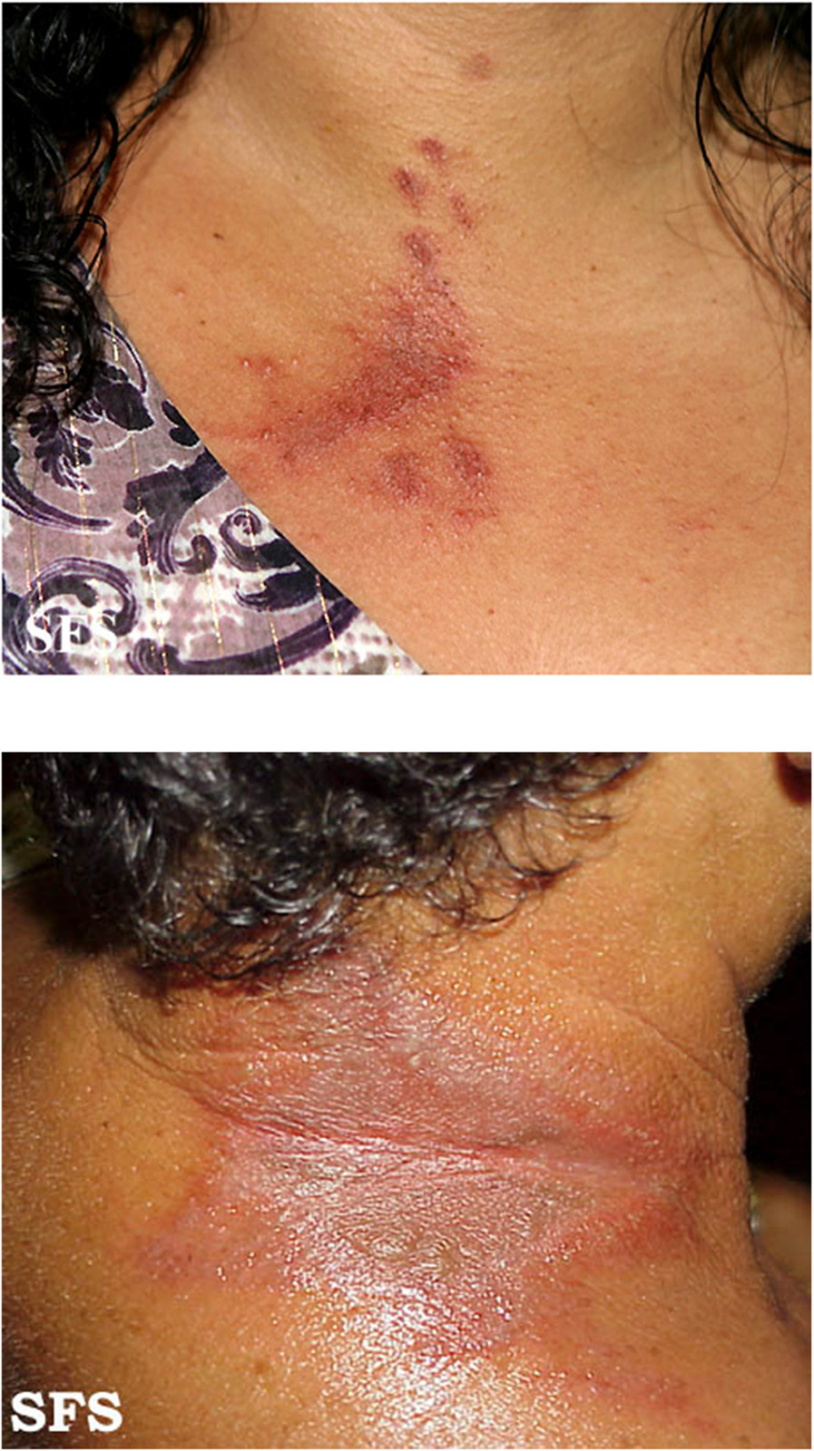
**General symptoms of** ***Paederus*** **dermatitis (images by Samuel Freire da Silva, M.D. in homage to the master and professor Delso Bringel Calheiros, available at** **http://www.atlasdermatologico.com.br/index.jsf** **).**
- Kissing lesions on the elbow and adjacent surfaces of the thighs due to contact of healthy areas with the infected ones [9].
- Sometimes respiratory, eye or skin allergies may be found [18].
- Pederin on hands and clothing/bed sheets can be transferred to and cause lesions on other areas (e.g., genitals and periorbital areas), though the dermatitis itself is not transferable.
- After 24 to 48 hours, the lesions may turn black or become infected.

Rove beetles are in offensive when we leave them unharmed on touching our body. If we try to dislodge them or kill them by crushing or smacking, they release a bodily fluid containing pederin on the exposed skin. The symptoms may appear after 24 to 48 hours of contact and take a week or more to disappear [6,18]. Although the severity of symptoms may depend upon the pederin concentration and contact duration on the body, the general symptoms are as follows:

- Swelling and burning sensation on the affected body part.
- Presence of linear reddish lesions giving a whiplash appearance on the neck as shown in Figure 3 [3,4].

These symptoms may be confused with herpes simplex, liquid burns, acute allergic or irritant contact dermatitis but we can arrive at the right diagnosis due to these epidemiological features (occurrence of similar cases in a given area, seasonal incidence and identification of the beetle) [9,18]. To avoid the spreading of symptoms, wash the affected area with clean, fresh water and soap and in case of eyes rinse them with fresh water. Most of the people use traditional methods (herbal creams and medicines locally made in homes) as compared to modern methods (antibiotics and pharmaceutical creams) for the treatment of this dermatitis, as shown in Table 5.

**Table 5:** **Methods applied for treatment of dermatitis**

**Patient’s locality**	**Traditional method (without modern medicines)**	**Modern methods**
Villages/small towns	91%	9%
Cities and adjoining areas	24%	76%

Traditional methods include herbal creams and medicines locally made in homes whereas modern methods include antibiotics and pharmaceutical creams.

On the basis of present study, the following preventive measures and treatment may be recommended to avoid this skin disease;

**Preventive Measures** (to avoid the human/beetle contact) [7,9]:

- Learn to identify the *Paederus* beetles.
- Avoid the crushing of these beetles on the exposed areas of the body.
- If a beetle crawls on your body, try to remove it gently by blowing it off or put a piece of paper on to which it may crawl and thus be removed.
- If you crush it or you think that you may have crushed the beetle while sleeping, take a shower and wash the cloths to avoid contact with pederin.
- As these insects are attracted towards light, turn off fluorescent lights or tie a net cloth under the light to avoid the dropping of these insects on the bed or human body [19].
- Check areas for the presence of insects near the light especially on walls or false ceiling before going to bed. If present, then kill them with insecticides or simply by beating with some object. Care should be taken for the removal of beetle carcasses and put them in a dustbin after putting them in plastic bags because they can cause symptoms in either an alive or dead form. Avoid handling them directly and washing hands after handling.
- Use screens on the doors and windows to prevent their entry.
- Repair the doors and windows if needed and tightly close the doors to avoid their entry.
- Try to sleep under a bed net to avoid their contact with the body at night in situations where a large population of these insects is present.
- Remove excessive vegetation from and around the home.

**Treatment:** the cases should be managed as follows [20]:

- Initially wash the affected area with soap and clean fresh water to remove the pederin.
- Application of cold wet compresses.
- Application of topical steroid and oral antihistamines.
- Use of oral antibiotics in case of secondary infection.

## Conclusions

The present study concludes that *Paederus* dermatitis is more prevalent in rural than urban areas due to the strong affinity (positive association) of rove beetles with crops and poor building conditions (broken doors and windows) [21-24]. Since the symptoms of this disease are mistaken for other skin diseases, the healthcare personnel need to be aware of the patient’s surrounding environment to achieve a correct diagnosis and treatment of this disease. This condition is generally prevalent when this insect is more active, i.e., in March and July-August.

### Ethics committee approval

The present study was approved by the Ethics Committee of the Government College University, Faisalabad, Pakistan.
